# Peripheral intravenous catheter failure, nurse staffing levels and care complexity individual factors: A retrospective multicentre cohort study

**DOI:** 10.1371/journal.pone.0303152

**Published:** 2024-05-09

**Authors:** Emilio Jiménez-Martínez, Jordi Adamuz, Maribel González-Samartino, Maria Antonia Muñoz-Carmona, Ana Hornero, M. Purificacion Martos-Martínez, Remedios Membrive-Martínez, Maria-Eulàlia Juvé-Udina

**Affiliations:** 1 Infectious Disease Department, Bellvitge University Hospital, L’Hospitalet de Llobregat (Barcelona), Barcelona, Spain; 2 Medicine and Health Science Faculty, School of Nursing, University of Barcelona, L’Hospitalet de Llobregat (Barcelona), Barcelona, Spain; 3 Bellvitge Institute of Biomedical Research, IDIBELL, Nursing Research Group, Barcelona, Spain; 4 Nursing Knowledge Management and Information Systems Department, Bellvitge University Hospital, L’Hospitalet de Llobregat (Barcelona), Barcelona, Spain; 5 Nursing Knowledge Management and Information Systems Department, Viladecans Hospital, Viladecans (Barcelona), Barcelona, Spain; 6 Catalan Institute of Health, Barcelona, Spain; Ain Shams University Faculty of Nursing, EGYPT

## Abstract

**Introduction:**

Short peripheral intravenous catheter (PIVC) failure is a common complication that is generally underdiagnosed. Some studies have evaluated the factors associated with these complications, but the impact of care complexity individual factors and nurse staffing levels on PIVC failure is still to be assessed. The aim of this study was to determine the incidence and risk factors of PIVC failure in the public hospital system of the Southern Barcelona Metropolitan Area.

**Methods:**

A retrospective multicentre observational cohort study of hospitalised adult patients was conducted in two public hospitals in Barcelona from 1^st^ January 2016 to 31^st^ December 2017. All adult patients admitted to the hospitalisation ward were included until the day of discharge. Patients were classified according to presence or absence of PIVC failure. The main outcomes were nurse staffing coverage (ATIC patient classification system) and 27-care complexity individual factors. Data were obtained from electronic health records in 2022.

**Results:**

Of the 44,661 patients with a PIVC, catheter failure was recorded in 2,624 (5.9%) patients (2,577 [5.8%] phlebitis and 55 [0.1%] extravasation). PIVC failure was more frequent in female patients (42%), admitted to medical wards, unscheduled admissions, longer catheter dwell time (median 7.3 vs 2.2 days) and those with lower levels of nurse staffing coverage (mean 60.2 vs 71.5). Multivariate logistic regression analysis revealed that the female gender, medical ward admission, catheter dwell time, haemodynamic instability, uncontrolled pain, communication disorders, a high risk of haemorrhage, mental impairments, and a lack of caregiver support were independent factors associated with PIVC failure. Moreover, higher nurse staffing were a protective factor against PIVC failure (AUC, 0.73; 95% confidence interval [CI]: 0.72–0.74).

**Conclusion:**

About 6% of patients presented PIVC failure during hospitalisation. Several complexity factors were associated with PIVC failure and lower nurse staffing levels were identified in patients with PIVC failure. Institutions should consider that prior identification of care complexity individual factors and nurse staffing coverage could be associated with a reduced risk of PIVC failure.

## Introduction

Short peripheral intravenous catheters (PIVC) are the most commonly used medical devices in hospitals, being widely applied in therapeutic support and diagnostic procedures. Although PIVC use is widespread, adverse events related to vascular catheterisation, such as catheter-related bloodstream infections and PIVC failure are frequent and are often overlooked [1,2]. PIVC failure is a common complication, with incidences ranging from 20% to 80% [3,4]. It is generally underdiagnosed and requires the replacement of the catheter in many cases [5–7]. This entails an additional invasive procedure, thus increasing patient discomfort, risks to health workers, and hospital costs [8–10]. Nowadays, the most serious complication of PVC failure, catheter related bloodstream infection, costs an estimated US$45,000 per event [11].

Previous studies have identified certain risk factors associated with PIVC failure such as female gender, age, comorbidities, the infusion of irritant drugs, type of catheter, circumference, and the site of access [12,13]. To date, few studies have evaluated the impact of other broad health determinants related to care complexity individual factors or nurse staffing levels [14,15]. Only one previous study showed a significant association between nurse staffing levels and phlebitis in patients admitted to intensive care units (ICU) [16]. Therefore, the impact of care complexity individual factors and nurse staffing levels on PIVC failure in hospitalised patients remains to be studied. In the context of this study, care complexity individual factors and nurse staffing coverage (acute to intensive care [ATIC] patient classification system) were implemented in nursing e-charts of Catalan public health hospitals as structured data based on the Architeture, Terminology, Interface, Knowledge terminology since 2013 [17]. Juvé-Udina et al. also identified care complexity individual factors (CCIF) in hospitalized patients, classifying them into five domains: developmental, mental-cognitive, psycho-emotional, sociocultural and comorbidity/complications,; four coincided with the Vector Model of Complexity that defines the determinants of complexity, along axes representing major health determinants. In this regard prior studies associated CCIF with health outcomes in hospitalized patients [18]. Concerning nurse staffing coverage, the ATIC patient classification system allows identification of patient acuity according to nursing care requirement. With this classification system, the necessary nursing coverage can be identified thanks to the result of the difference between required and available nursing hours [19]. Although, a few studies show the impact of CCIF and nursing coverage in health outcomes [20,21], nowadays research focusing on phlebitis and PIVC failure is still needed.

### Aim

Since quality evidence regarding PIVC failure, its risk factors, incidence, and prevention is still required, the aim of this study was to determine the incidence and risk factors associated with PIVC failure in the public hospital system of the Southern Barcelona Metropolitan Area.

## Methods

### Study design, setting, and patients

A retrospective multisite observational cohort study of hospitalised adult patients was conducted with data collected at two public hospitals (a teaching tertiary referral third-level hospital and a second-level hospital) in the Southern Barcelona Metropolitan Area, part of the *Institut Català de la Salut*, from 1^st^ January 2016 to 31^st^ December 2017. These hospitals serve an area with 1,100,000 inhabitants and admit approximately 50,000 patients per year.

All adult patients admitted to the inpatient wards of these hospitals during the study period with a completed hospital minimum data set report were included from the day of admission until the day of discharge. Obstetrics, maternal-child, and paediatric patients were excluded. All PIVC and PIVC-related events during pre-pandemic period were retrospectively followed up. Data were obtained retrospectively from electronic health records, the hospital minimum data set and the clinical data warehouse of the Catalan Institute of Health in 2022. With a unique identification number, data sets were linked. After merging the databases, the quality and adequacy of patient data was assessed. The study was intended to include all consecutively admitted patients matching the selection criteria. This represented an initial sample estimation of 47,249 adults.

### Main outcomes, variables, and data source

Patients were classified according to presence or absence of PIVC failure. PIVC failure was defined as an unscheduled dysfunction of a vascular catheter due to phlebitis, thrombosis, extravasation, or a suspected infection [22]. Data were collected from the electronic nurse records, where registered nurses reported during patient admission any of the following nurse diagnoses: catheter-associated phlebitis (code: 10001284) and extravasation (code: 10002222), according to ATIC terminology. The surveillance of PIVC was made by nurses daily, 8 hourly according to the *Institut Català de la Salut* protocols [23]. The following basic data on demographics as well as information on patient comorbidities and their clinical and health outcomes were collected: gender, age, length of stay, scheduled admission, catheter dwell time, nurse staffing and workforce measures, and care complexity individual factors.

Information on nurse staffing and workforce measures included available registered nurse (RN) hours, required RN hours, and RN staffing coverage. Available RN hours per patient day (*a*NHPPD) were obtained by dividing the available RN hours by the total number of patients in the units every day before being aggregated into unit-shift and unit-day levels. The required RN hours per patient day (*r*NHPPD) were measured with the ATIC patient classification system, which clusters acuity into ten categories of nursing intensity that is equivalent to the required RN hours per patient day. This data was recruited in nursing e-charts based on patient acuity identified by the weight of each main nursing diagnosis. Nurse staffing coverage was defined as the proportion of RN *r*NHPPD reached by the *a*NHPPD [15]. A recent study considered that a staffing level below 80% of the median level in the units constituted understaffing [24]. Nursing assistants and other support staff were not considered.

Care complexity individual factors are defined as a set of characteristics related to different health dimensions that have the potential to increase difficulties in the process of care delivery and raise healthcare utilisation [25,26]. Care complexity individual factors can be divided into five domains: (a) developmental, (b) mental-cognitive, (c) psycho-emotional, (d) sociocultural, and (e) comorbidity or complications. Each domain is structured into factors and specifications. Patients were considered to fall within any of the domains of the care complexity individual factors if they presented at least one specification during their hospitalisation. The specifications of the care complexity individual factors were related to the nursing assessment elements based on the terminology of Architecture, Terminology, Interface, and Knowledge (ATIC in Catalan spelling) (Juvé-Udina, 2013) and recorded in the electronic charts.

### Statistical methods

Quantitative variables are reported as the mean, median, standard deviation, and interquartile range (IQR), while categorical variables are reported as absolute numbers and percentages. To detect significant differences between the groups, chi-square test or Fisher’s exact test was used for the categorical variables, while Student’s *t-*test or the Mann–Whitney *U* test was applied for the continuous variables, as appropriate. The factors associated with PIVC failure were evaluated by univariate and multivariate analyses. The logistic regression model of the factors potentially associated with PIVC failure included all the significant care complexity individual factors detected in the univariate analysis, the catheter dwell time, the nursing coverage, and possible confounders (gender, medical ward admission, and ICU admission). This analysis was adjusted by the type of hospital (third-level or second-level hospital). The discriminatory power of the final multivariate model was assessed by the area under the receiver operating characteristic curve (AUROC). The results of the multivariate analyses are reported as the odds ratios (OR) and 95% confidence intervals (CI). Statistical analysis was performed with version 25.0 of the SPSS software package (SPSS, Chicago, IL, USA). Statistical significance was established at α = 0.05, and all reported *p*-values are two-tailed.

### Ethics

The need for informed consent and the provision of an information sheet were waived because of the retrospective nature of the study. Ethical standards related to data anonymity and confidentiality (access to records, data encryption, and archiving of information) were observed throughout the research process. Confidential information of the patients was protected, in compliance with European regulations.

The Clinical Research Ethics Committee of Bellvitge University Hospital approved the study (PR243/20).

This paper was written in accordance with the STROBE statement (https://strobe-statement.org/index.php?id=available-checklists).

## Results

During the study period, 47,249 adult patients were admitted to the hospital wards and 44,661 (94.5%) had a PIVC inserted during hospitalisation. PIVC failure was recorded in 2,624 (5.9%) hospitalised patients (2,577 [5.8%] phlebitis and 55 [0.1%] extravasation). Around 42% of patients were female, with a median age of 68 years. Half of the patients were admitted unscheduled to the medical wards. The median hospital stay was four days: almost 3% were admitted to the ICU and 13% to step-down units.

### Patient characteristics

The demographic and clinical characteristics of the patients with and without PIVC failure are compared in Table 1. Patients with PIVC failure were more often female, older, admitted to the medical wards, the step-down units or ICU, and their admission was more likely to be unscheduled. Moreover, patients with longer hospital stays experienced higher rates of PIVC failure. Likewise, PIVC failure was more common in patients with a longer catheter dwell time (median 7.3 vs 2.2 days). Furthermore, PIVC failure was more frequently identified in patients admitted for infectious disease or for digestive, liver, pancreatic or nervous system issues.

**Table 1:** Baseline characteristics of adult patients according to PIVC failure.

Characteristics	Study population n = 44,661		PIVC failure n = 2,624 (5.9)		PIVC without failure n = 42,037 (94.1)		p-Value
	No.	(%)	No.	(%)	No.	(%)	
Age (years)_median (IQR)	68	(54–77)	70	(57–80)	67	(54–77)	<0.001
Female sex	19,013	(42.6)	1,175	(44.8)	17,838	(42.4)	0.019
Medical ward	22,827	(51.1)	1,811	(69.0)	21,016	(50.0)	<0.001
Surgical ward	21,834	(48.9)	813	(31.0)	21,021	(50.0)	<0.001
Step-down unit	5,269	(11.8)	423	(16.1)	4,846	(11.5)	<0.001
ICU admission	1,146	(2.6)	93	(3.5)	1,053	(2.5)	0.001
Unscheduled admission	22,147	(49.6)	1,996	(76.1)	20,151	(47.9)	<0.001
Length of stay_median (IQR)	4	(1–8)	10	(6–16)	4	(1–7)	<0.001
Catheterization dwell days _ median (IQR)	2.4	(1.0–5.6)	7.3	(4.1–12.7)	2.2	(1.0–5.1)	<0.001
**Reason for admission**							
Cardiocirculatory	8,098	(18.1)	376	(14.3)	7,722	(18.4)	<0.001
Infectious	7,055	(15.8)	714	(27.2)	6,341	(15.1)	<0.001
General surgery	5,064	(11.3)	175	(6.7)	4,889	(11.6)	<0.001
Trauma and orthopaedics	4,220	(9.4)	142	(5.4)	4,078	(9.7)	<0.001
Digestive, liver, and pancreatic	5,823	(13.0)	484	(18.4)	5,339	(12.7)	<0.001
Nervous system	3,344	(7.5)	287	(10.9)	3,057	(7.3)	<0.001
Kidney and urinary tract	3,573	(8.0)	171	(6.5)	3,402	(8.1)	0.004
Respiratory	2,013	(4.5)	113	(4.3)	1,900	(4.5)	0.66
Reproductive	1,615	(3.6)	22	(0.8)	1,593	(3.8)	<0.001
Head, neck and maxillofacial	1,501	(3.4)	55	(2.1)	1,446	(3.4)	<0.001
Metabolic, nutritional and endocrinology	821	(1.8)	15	(0.6)	802	(1.9)	<0.001
Hematopoiesis, blood and immunologic	451	(1.0)	31	(1.2)	420	(1.0)	0.21
Psychiatric, mental health and addictions	37	(0.1)	2	(0.1)	35	(0.1)	0.63
Skin and burns	56	(0.1)	4	(0.2)	52	(0.1)	0.57
Eyes	476	(1.1)	2	(0.1)	474	(1.1)	<0.001
Other	514	(1.2)	31	(1.2)	483	(1.1)	0.86

Abbreviations: IQR, interquartile range.

### Nurse staffing measures and care complexity individual factors

As shown in Table 2, the available NHPPD was similar in the patients with PIVC failure and in those without (2.8 vs 2.7 respectively), while the required NHPPD was higher in the patients with PIVC failure (5.2 vs 4.3, respectively). Thus, PIVC failure was more frequent in patients with lower levels of nurse staffing coverage (mean 60.2 in patients with PIVC failure vs 71.5 in patients without PIVC failure).

**Table 2:** Nursing staffing measures and care complexity individual factors of admitted patients according to PIVC failure.

Characteristics	Study population n = 44,661		PIVC failure n = 2,624 (5.9)		PIVC without failure n = 42,037 (94.1)		
	No.	%	No.	%	No.	%	*p value*
**Staffing and workforce measures**							
aNHPPD_mean (SD)	2.7	(1.0)	2.8	(1.1)	2.7	(1.0)	<0.001
rHPPD_mean (SD)	4.4	(1.7)	5.2	(1.7)	4.3	(1.7)	<0.001
Balance_mean (SD)	-1.7	(2.0)	-2.3	(2.0)	-1.6	(2.0)	<0.001
Nurse staffing coverage_% mean (SD)	70.9	(36.8)	60.2	(29.6)	71.5	(37.1)	<0.001
***Care complexity individual factors (CCIF)***							
* * ***Comorbidity/complications***							
Transmissible infection	1,541	(3.5)	192	(7.3)	1,349	(3.2)	<0.001
Hemodynamic instability	26,484	(59.3)	1,861	(70.9)	24,623	(58.6)	<0.001
Chronic disease	19,641	(44.0)	1,319	(50.3)	18,322	(43.6)	<0.001
Uncontrolled pain	11,274	(25.2)	987	(37.6)	10,287	(24.5)	<0.001
Extreme weight	2,442	(5.5)	170	(6.5)	2,272	(5.4)	0.02
Position impairment	2,051	(4.6)	119	(4.5)	1,932	(4.6)	0.92
Urinary or fecal incontinence	4,351	(9.7)	337	(12.8)	4,014	(9.5)	<0.001
Immunosuppression	241	(0.5)	24	(0.9)	217	(0.5)	0.01
Anatomical and functional disorders	5,121	(11.5)	334	(12.7)	4,787	(11.4)	0.04
Communication disorders	1,919	(4.3)	231	(8.8)	1,688	(4.0)	<0.001
High risk of hemorrhage	1,403	(3.1)	120	(4.6)	1,283	(3.1)	<0.001
Vascular fragility	1,753	(3.9)	129	(4.9)	1,624	(3.9)	0.008
Involuntary movements	209	(0.5)	21	(0.8)	188	(0.4)	0.02
Dehydration	17	(0.0)	2	(0.1)	15	(0.1)	0.26
Edema	753	(1.7)	65	(2.5)	688	(1.6)	0.002
* * ***Developmental***							
Old age (≥75 years)	14,075	(31.5)	992	(37.8)	13,083	(31.1)	<0.001
* * ***Psycho-emotional***							
Fear/anxiety	5,856	(13.1)	345	(13.1)	5,511	(13.1)	0.95
Impaired adaptation	2,523	(5.6)	204	(7.8)	2,319	(5.5)	<0.001
Aggressive behavior	134	(0.3)	8	(0.3)	126	(0.3)	0.86
* * ***Mental-cognitive***							
Mental status impairments	3,065	(6.9)	318	(12.1)	2,747	(6.5)	<0.001
Agitation	395	(0.9)	40	(1.5)	355	(0.8)	<0.001
Impaired cognitive functions	110	(0.2)	7	(0.3)	103	(0.2)	0.84
Perception of reality disorders	69	(0.2)	6	(0.2)	63	(0.1)	0.3
* * ***Sociocultural***							
Lack of caregiver support	1,279	(2.9)	131	(5.0)	1,148	(2.7)	<0.001
Belief conflict	70	(0.2)	4	(0.2)	66	(0.2)	0.61
Language barriers	117	(0.3)	11	(0.4)	106	(0.3)	0.11
Social exclusion	28	(0.1)	1	(0.1)	26	(0.1)	0.68

Abbreviations: SD, standard deviation; aNHHPD, Available RN hours per patient day; rNHPPD, Required RN hours per patient day.

Regarding the comorbidity/complications domain of the care complexity individual factors, transmissible infections, haemodynamic instability, chronic diseases, uncontrolled pain, extreme body weight, mixed incontinence, immunosuppression, anatomical and functional disorders, a high risk of haemorrhage, vascular fragility, involuntary movements, and oedema were more frequently identified in patients with PIVC failure than in those without. Concerning the other domains of care complexity individual factors, PIVC failure was more common in older patients, those with impaired adaptations, those with mental impairments, those with agitation, and those lacking caregiver support (Table 2).

### Risk factors associated with PIVC failure

The results of the multivariate logistic regression analysis for all the care complexity individual factors and other demographic and clinical characteristics potentially associated with PIVC failure are summarised in Table 3. After adjusting for the type of hospital, the multivariate logistic regression analysis of PIVC failure showed independent associations with female gender (odds ratio [OR]: 1.17; 95% confidence interval [CI]: 1.08–1.27), medical ward admission (OR: 1.61; 95% IC: 1.46–1.79), catheter dwell time (OR: 1.10; 95% IC: 1.09–1.10), haemodynamic instability (OR: 1.23; 95% IC: 1.12–1.35), uncontrolled pain (OR: 1.33; 95% IC: 1.21–1.46), communication disorders (OR: 1.43; 95% IC: 1.21–1.70), a high risk of haemorrhage (OR: 1.39; 95% IC: 1.13–1.70), mental impairments (OR: 1.18; 95% IC: 1.00–1.38), and a lack of caregiver support (OR: 1.17; 95% IC: 1.05–1.30). Moreover, a higher level of nurse staffing coverage (OR: 0.99; 95% IC: 0.99–0.99) was a protective factor against PIVC failure. The area under the ROC curve was 0.73 (95% CI: 0.72–0.74).

**Table 3:** Multivariate analysis of clinical characteristics in adult hospitalized patients associated with PIVC failure.

Clinical characteristics	PIVC failure			*p value*
	n = 2,624 (5.9)			
	OR	(95% CI)		
Female sex	1.171	1.076–1.275	<0.001
Medical ward	1.614	1.457–1.788	<0.001
ICU admission	0.924	0.720–1.186	0.535
Nurse staffing coverage	0.995	0.993–0.997	<0.001
Catheterization dwell days	1.097	1.091–1.103	<0.001
Transmissible infection	1.057	0.884–1.265	0.542
Hemodynamic instability	1.231	1.120–1.353	<0.001
Chronic disease	0.943	0.864–1.030	0.191
Uncontrolled pain	1.327	1.210–1.456	<0.001
Extreme weight	0.972	0.818–1.154	0.743
Urinary or fecal incontinence	0.968	0.840–1.116	0.656
Immunosuppression	1.274	0.815–1.991	0.289
Anatomical and functional disorders	0.997	0.877–1.133	0.961
Communication disorders	1.435	1.211–1.700	<0.001
High risk of hemorrhage	1.389	1.134–1.701	0.001
Vascular fragility	0.981	0.802–1.199	0.848
Involuntary movements	1.042	0.635–1.709	0.871
Edema	0.945	0.713–1.251	0.691
Old age (≥75 years)	0.980	0.892–1.077	0.674
Impaired adaptation	0.906	0.765–1.074	0.256
Mental status impairments	1.177	1.003–1.381	0.046
Agitation	0.879	0.609–1.268	0.490
Lack of caregiver support	1.169	1.048–1.305	0.005

Abbreviations: OR, odds ratio; CI, confidence interval.

AUC: 0.730 (IC 95% 0.720–0.741).

## Discussion

In this study of a large number of hospitalised patients, we found that almost 6% experienced PIVC failure (phlebitis or extravasation). The risk factors independently associated with PIVC failure were female gender, medical ward admission, catheter dwell time, haemodynamic instability, uncontrolled pain, communication disorders, a high risk of haemorrhage, mental impairments, and a lack of caregiver support. A higher nurse staffing level could be associated with a reduced risk of PIVC failure.

Around 6% of admitted patients experienced phlebitis or extravasation, which is similar to the rates of around 4%-19% reported previously [4,12,13]. However, those studies presented substantial variations in the inclusion criteria, PIVC failure definitions and marked differences in patients’ baseline characteristics.

Medical ward admission, female gender, and catheter dwell time were independent factors associated with PIVC failure. In agreement with previous reports that patients admitted to medical wards have higher rates of phlebitis, pressure ulcers and falls [27], the findings of our study showed that medical ward admission was an independent factor associated with PIVC failure.

A few studies have found an association between gender and PIVC failure. These findings may be related to the smaller calibre of blood vessels in females compared to males, causing more endothelial damage [13]. Moreover, some researchers suggest that different hormonal and adipose tissue distributions can influence PIVC failure [28]. More studies are needed to support this hypothesis due to the under-representation of women in clinical trials testing medical devices, which makes it difficult to determine the efficacy of devices in female patients.

Catheter dwell time was longer in patients with PIVC failure. Our results are in line with those of several studies demonstrating that the risk of PIVC failure is directly related to the dwell time [7,12,29]. Although evidence indicates that a PIVC should not be used for longer than 7 days [30], there are several factors that affect the replacement with a new vascular catheter. Firstly, the clinical condition of the patient might not allow for a catheter replacement at the right time. Secondly, much of the vascular capital of patients is damaged by treatment and the insertion of a new PIVC may be difficult.

To the best of our knowledge, this is the first study to identify the impact of broader health determinants (measured by the care complexity individual factors) and nurse staffing levels on PIVC failure. After adjusting for potential confounders and the type of hospital in the multivariate analysis, we found that several care complexity individual factors and nurse staffing levels were associated with PIVC failure.

Haemodynamic instability, uncontrolled pain, and a high risk of haemorrhage were associated with PIVC failure. Patients admitted with haemodynamic instability, uncontrolled pain, and a high risk of haemorrhage usually receive fluid infusion. In this regard, previous studies have shown that infusing irritant drugs is an independent factor associated with PIVC complications such as infiltration, occlusion, and phlebitis associated with intravenous medications [13,31,32]. In fact, irritant fluid infusion, daily infusion time, daily infusion volume, and the type of sealing liquid were recently reported to be independent predictors of PIVC failure [33]. Irritant fluids and drugs, such as antibiotics, vasoactive drugs, antihaemorrhagic drugs, and dexamethasone, have an impact on PIVC failure [7,34]. A previous study suggested that high concentrations of irritant fluids can increase the plasma osmotic pressure, cause a fluid shift from within the vascular endothelial cells to the extracellular space, elicit infiltration or extravasation, and produce vascular stiffness [33].

Mental impairments, communication disorders, and a lack of caregiver support were also identified as independent factors associated with PIVC failure. Patients with mental impairments are more likely to present confusion, disorientation, stupor, or a transient loss of consciousness that may have an impact on the clinical outcomes of hospitalised patients [14]. Additionally, previous studies have shown that patients admitted to neurology or internal medicine wards with a peripheral venous catheter present a higher frequency of delirium [35], which can be associated with PIVC failure. Furthermore, the accidental removal of devices is more likely to occur in patients requiring intensive care [36] and those who frequently present confusion and communication disorders [37]. Finally, previous studies have also found that a lack of caregiver support during hospitalisation is associated with adverse events. In this line, previous findings report that unpaid caregivers often take on an active caregiving role in hospitals to mitigate the risk of functional decline and hospital-related adverse events [14,38].

The nurse staffing level is proportionally related to the supply of quality care. It has been shown that nurse understaffing is associated with an increased risk of healthcare-associated infections, adverse events, and mortality [39,40]. To the best of our knowledge, only one previous study in intensive care settings demonstrated an association between catheter-related phlebitis and the mean number of nursing care hours [16]. Therefore, this is the first study to association of nurse staffing coverage with PIVC failure in hospitalised patients, although the results were statistically significant shown a protective effect of nursing staffing coverage, we obtained a poor association. This data concurs with previous studies that identified a lower nursing coverage and poor health outcomes in patients admitted in general wards [20]. Our study detected a lower nurse staffing level in patients with PIVC failure (60.2% vs. 71.5% from those who did not PIVC failure). It should be noted that previous studies considered units to be understaffed when they were below 80% of the median nursing staff coverage [41]. Therefore, nurse staffing coverage should be considered in healthcare policy.

### Limitations

The strengths of this study include its multicentre observational cohort design involving a large number of patients. Also, it is the first study to evaluate the impact of nurse staffing levels and broader health conditions on PIVC failure in hospitalised patients. However, there are also some limitations that should be acknowledged. All the data were comprehensively collected from electronic health records that included nursing assessments at the time of admission and were re-evaluated during hospitalisation. Therefore, we assumed proper compliance with electronic health records. However, they might have contained coding errors. We did not consider other factors that might contribute to PIVC failure, such as catheter gauges, infusion of intravenous antibiotics or other fluids, and the site of catheter insertion. Moreover, although we collected all the PIVC failures recorded in the health records, we did not collect information regarding grade of phlebitis and other complications such as dislodgement and occlusion. Finally, the results show a fair overall model, therefore future studies should corroborate these findings.

## Conclusion

Almost 6% of patients experienced PIVC failure during hospitalisation. The risk factors independently associated with PIVC failure were female gender, medical ward admission, catheter dwell time, haemodynamic instability, uncontrolled pain, communication disorders, a high risk of haemorrhage, mental impairments, and a lack of caregiver support. Lower nurse staffing levels were identified in patients with PIVC failure. Therefore, a higher nurse staffing level could be associated with a reduced risk of PIVC failure. Institutions should consider that prior identification of care complexity individual factors and nurse staffing coverage could be associated with a reduced risk of PIVC failure. Future prospective studies should support these findings including other PIVC complications.
